# Combined Supplementation of Choline and Docosahexaenoic Acid during Pregnancy Enhances Neurodevelopment of Fetal Hippocampus

**DOI:** 10.1155/2017/8748706

**Published:** 2017-01-22

**Authors:** Huban Thomas Rajarethnem, Kumar Megur Ramakrishna Bhat, Malsawmzuali Jc, Siva Kumar Gopalkrishnan, Ramesh Babu Mugundhu Gopalram, Kiranmai Sesappa Rai

**Affiliations:** ^1^Department of Anatomy, Kasturba Medical College, Manipal University, Manipal, India; ^2^Department of Physiology, Kasturba Medical College, Manipal University, Manipal, India; ^3^Department of Physiology, Melaka-Manipal Medical College, Manipal University, Manipal, India

## Abstract

Choline is an essential nutrient for humans which plays an important role in structural integrity and signaling functions. Docosahexaenoic acid (DHA) is a polyunsaturated fatty acid, highly enriched in cell membranes of the brain. Dietary intake of choline or DHA alone by pregnant mothers directly affects fetal brain development and function. But no studies show the efficacy of combined supplementation of choline and DHA on fetal neurodevelopment. The aim of the present study was to analyze fetal neurodevelopment on combined supplementation of pregnant dams with choline and DHA. Pregnant dams were divided into five groups: normal control [NC], saline control [SC], choline [C], DHA, and C + DHA. Saline, choline, and DHA were given as supplements to appropriate groups of dams. NC dams were undisturbed during entire gestation. On postnatal day (PND) 40, brains were processed for Cresyl staining. Pups from choline or DHA supplemented group showed significant (*p* < 0.05) increase in number of neurons in hippocampus when compared to the same in NC and SC groups. Moreover, pups from C + DHA supplemented group showed significantly higher number of neurons (*p* < 0.001) in hippocampus when compared to the same in NC and SC groups. Thus combined supplementation of choline and DHA during normal pregnancy enhances fetal hippocampal neurodevelopment better than supplementation of choline or DHA alone.

## 1. Introduction

Human brain development begins in the third gestational week and is a protracted process that depends on the differentiation of neural progenitor cells and continues unto late adolescence, impacting brain functions for the entire lifespan [1]. Hippocampus is a part of the brain which forms the limbic system and is important for emotional learning [2]. The role of the hippocampus in relational memory is that it receives multiple inputs to create and allows for storage of representations of the associations among the constituent elements of scenes and events [3]. This function of hippocampus is important for the long-term memory in cortical regions of the brain. The hippocampus develops reciprocal connections with cortex through medial temporal lobe. The hippocampal formation consists of the dentate gyrus, cornu ammonis (CA) regions, and the subiculum. The dentate gyrus receives afferent connections from the entorhinal cortex, which act as an input region. The cornu ammonis regions of the hippocampus are comprised mainly of pyramidal cells [4–6]. The entorhinal cortex and the CA1 region of the hippocampus establish reciprocal connections via the subiculum [7]. This reciprocal connection involves two pathways, one between the dentate gyrus and CA3 region of the hippocampus and the other between the CA1 region of the hippocampus and subiculum. The subiculum intern sends an efferent input back to the entorhinal cortex [8]. The hippocampus in humans develops during the last trimester and the volume of hippocampus is fully matured at 15 months of age, whereas in rats hippocampus develops during embryonic [E] days E11–E17 [9].

Neurodevelopment of fetal brain is influenced by various dietary nutrients including essential nutrients such as choline and docosahexaenoic acid (DHA). Choline is the major source of methyl groups in the diet; it is needed for the structural integrity and signaling functions of cell membranes. Choline concentration directly affects cholinergic neurotransmission, transmembrane signaling, and lipid transport as well as metabolism [10]. Dietary choline concentration influences neural tube closure, hippocampal development, apoptotic signaling in neurons and in liver cells, hepatic transport of lipoproteins, and hepatic carcinogenesis [11]. Moreover choline and DHA are related by the influence of phosphatidylethanolamine-*N*-methyltransferase (PEMT) that catalyzes the biosynthesis of phosphatidylcholine [PtdCho] from phosphatidylethanolamine which is enriched in long-chain polyunsaturated fatty acids, such as arachidonic acid and docosahexaenoic acid [12, 13]. Observations from recent studies indicate that DHA is necessary for neurodevelopment, neurotransmission, and protecting the brain from oxidative stress. These functions explain the important role of DHA within the core of neural membranes [14]. DHA maintains the activity of Na+/K+ ATPase, which is the key cell membrane enzyme that draws out energy from ATP to drive the cellular sodium pump, which is necessary to controls the electrical impulses between the cells [15]. Phosphatidylserine (PS) is an important component of the cell membrane and is vital for cell survival; its concentration in brain is regulated by DHA, and thereby DHA influence the cognitive process [16]. DHA deficiency causes reduction in brain PS, which affects cell signaling for survival through enzymes such as Na+/K+ ATPase and by calcium uptake. Calcium is one of the multifaceted and most common among cell-signaling systems and is regulated by DHA [17]: DHA regulate a vast array of cellular functions, including function of the mitochondria, activation of gene, neurotransmitter release, developing brain, and neuron maturation and migration [18].

Alternately, various studies show that choline deficiency during fetal development reduces proliferation and migration of neuronal precursor cells in the mouse fetal hippocampus and these changes are associated with modifications in the protein levels of some cell cycle regulators [19]. When rodents are exposed to choline deficiency during their fetal development, they show decreased cell division and increased apoptosis in their hippocampus [20–22]. Choline-deficient diets fed to dams increased the release of the following markers and regulators of neural proliferation and differentiation, namely, TGF_1, a growth factor; calretinin, marker for early neuronal differentiation; p27Kip1, known for cyclin-dependent kinase inhibitor; and TOAD-64, which is the marker for neuronal differentiation, in the fetal brain [23–25]. Various animal studies have clearly shown that DHA deficiency during embryogenesis and subsequently during lactation could not be fully corrected later in life [26]. DHA status during birth has long-term impact and DHA deficiency was found to affect the behaviors in children until 7 years of age [27]. The present study was designed to analyze the efficacy of either combined or individual supplementation of choline and DHA during gestation on fetal hippocampal and dentate gyrus neurodevelopment in rats.

## 2. Materials and Methods

The present study was carried out in accordance with guidelines laid by Manipal University Institutional Animal Ethics Committee (IAEC/KMC/32/12), Manipal, India. Laboratory rats of Wistar strain, weighing approximately 200 g, were housed in Central Animal Research Facility, Manipal University, Manipal. Rats were housed in polypropylene cages containing sterile paddy husk as bedding and maintained under standard controlled lab conditions with temperatures ranging 23 ± 2°C, humidity (50 ± 5%), and 12 h light-dark cycle. All animals were fed with rat pellet feed obtained from (with choline content 1 mg/kg feed) VRK Nutritional Solutions, VRK's “Scientist's Choice” Laboratory Animal Diets Pune – 411037 (Table 1) and allowed free access to water ad libitum. Female rats in estrus cycle were identified and allowed for mating with male rats at 2 : 1 ratio. The first day of gestation was determined by the vaginal smear test. Once pregnancy was confirmed pregnant female rats were isolated and provided with nesting material and allowed to litter naturally.

Rat pellet feed fatty acid composition was analyzed by National Institute of Nutrition, Indian Council of Medical Research, Hyderabad, India (Table 2).

## 3. Study Design

Pregnant dams confirmed by vaginal smear test were divided into five groups: (1) normal control [NC], (2) saline control [SC], (3) choline [C], (4) docosahexaenoic acid [DHA], and (5) choline and DHA [C + DHA] groups. Pregnant dams from NC group were undisturbed in their home cage and were provided with normal animal feed and water ad libitum. Pregnant dams from saline control group were supplemented with saline throughout the period of gestation [from embryonic day 0 (E0) to delivery], choline group were supplemented with choline chloride throughout the period of gestation [choline chloride 98% Extra Pure obtained from Loba Chemie Laboratory Reagents and Fine Chemicals] dissolved with distilled water (4.6 mmol/kg/day of choline) [28], DHA group were supplemented with DHA throughout the period of gestation [soft gelatin capsules containing 300 mg docosahexaenoic acid/capsule were obtained from Nouveau Medicament (P) Ltd., Chennai] as such from the capsules (400 mg/day of DHA) [29], and choline + DHA group were supplemented with both choline and DHA throughout the period of gestation. Supplementation of the components stated previously was given to the rats by feeding needles. Following gestation dams were allowed to deliver naturally and pups from each group of dams were maintained undisturbed in their home cages for a period of 40 days.

Pups on the 40th day were anaesthetized by ether and the brain was perfused transcardially with saline followed by 10% formalin. Brain was excised and processed for paraffin sectioning. 300 sections of 5 *μ* thickness of the entire hippocampus were obtained. One section/30 hippocampal sections was serially selected and a total of 10 sections from each rat were stained with Cresyl Violet staining method. Sections were observed under light microscope at 40x magnification. Neural cell count was carried out by another experimenter, blind to the study, after coding the slides. 250 *μ*m area was randomly selected in CA1, CA3, and CA4 subregions as well as upper blade of dentate gyrus from each of the selected 5 *μ* thick hippocampal section and unbiased quantification was done manually using ocular micrometer scale. Data were analyzed using one-way ANOVA followed by Tukey's multiple comparison tests and expressed as mean ± SEM with the significance level at *p* < 0.05 using statistical software Graph pad prism version 5.03.

## 4. Results

**Figure 1:**
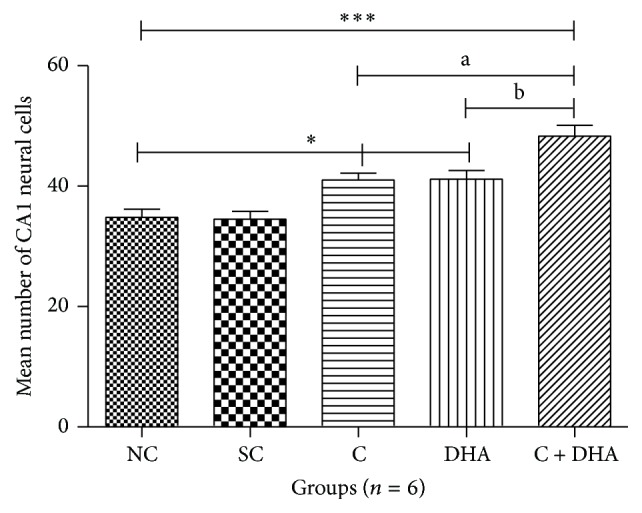
Comparison of numbers of neural cells in CA1 region in 5 *μ* thick hippocampal sections. Mean number of neural cells ± Standard Error from 250 *μ*m area of CA1 region of hippocampus from either side in normal control (NC), saline control (SC), choline (C), docosahexaenoic acid (DHA), and choline + docosahexaenoic acid (C + DHA) age matched group of rats. One-way ANOVA followed by Tukey's post hoc test. “*∗*”, NC versus supplemented groups; “a”, C supplemented group versus C + DHA group; and “b”, DHA supplemented group versus C + DHA group. ^*∗*/a/b^*p* < 0.05  &  ^*∗∗∗*^*p* < 0.001.

**Figure 2:**
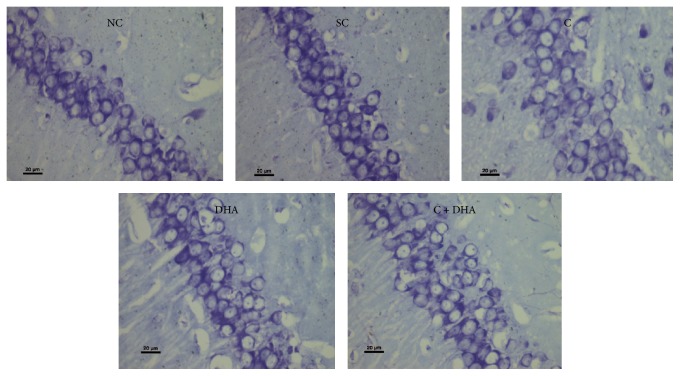
CA1 region: representative photomicrographs of CA1 region from 5 *μ* thick hippocampal sections viewed by 40x magnification, showing Cresyl Violet stained neurons from all experimental groups of PND 40 rats. Note: rats supplemented with combined choline and DHA show significant increase in the number of neural cells in CA1 region.

PND 40 rats supplemented prenatally with either choline or DHA alone show significant (^*∗*^*p* < 0.05) increase in the number of neural cells when compared with the same in age matched normal control and saline control group of rats [Figures 1 and 2]. However, PND 40 rats supplemented prenatally with choline + DHA showed significantly (^*∗∗∗*^*p* < 0.001) higher number of neural cells when compared with the same in age matched normal control and saline control group of rats [Figures 1 and 2]. Additionally, significantly higher number [^a, b^*p* < 0.05] of hippocampal neural cells in CA1 subregion was observed when compared with the same in age matched rat groups supplemented with either choline or DHA alone [Figures 1 and 2].

**Figure 3:**
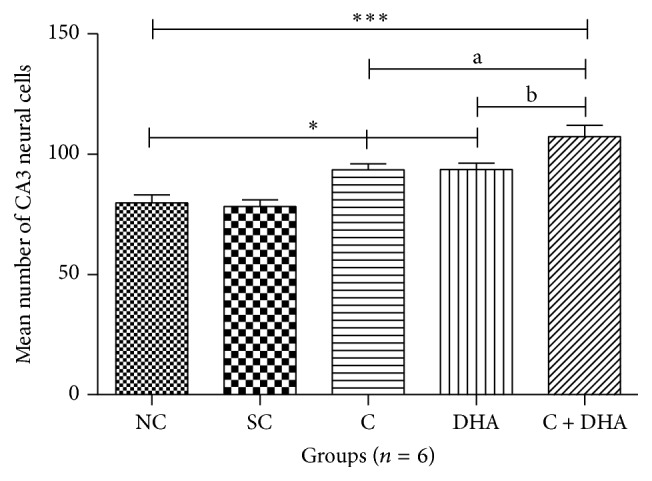
Comparison of neural cells in CA3 region of 5 *μ* thick hippocampal sections. Mean number of neural cells ± Standard Error from 250 *μ*m area of CA3 region of hippocampus from either side in normal control (NC), saline control (SC), choline (C), docosahexaenoic acid (DHA), and choline + docosahexaenoic acid (C + DHA) age matched group of rats. One-way ANOVA followed by Tukey's post hoc test. “*∗*”, NC versus supplemented groups; “a”, C supplemented group versus C + DHA group; and “b”, DHA supplemented group versus C + DHA group. ^*∗*/a/b^*p* < 0.05  &  ^**∗∗∗**^*p* < 0.001.

**Figure 4:**
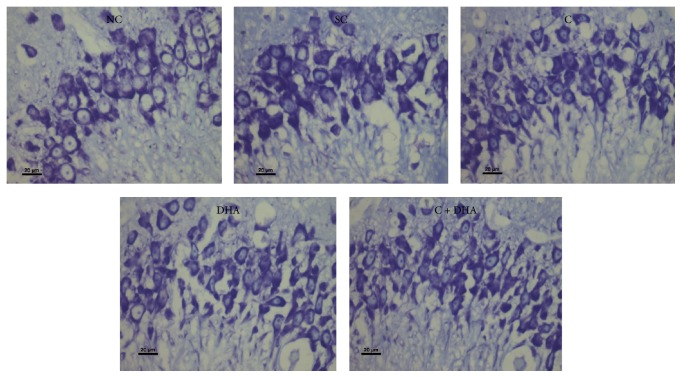
CA3 region: representative photomicrographs of CA3 region from 5 *μ* thick hippocampal sections viewed by 40x magnification, showing Cresyl Violet stained neurons from all experimental groups of PND 40 rats. Note: rats supplemented with combined choline and DHA show significant increase in the number of neural cells in CA3 region.

PND 40 rats supplemented prenatally with either choline or DHA alone shows significantly (^*∗*^*p* < 0.05) increased number of neural cells in the CA3 region of hippocampus when compared to the same in age matched normal control and saline control group of rats. However, PND 40 rats supplemented prenatally with both choline + DHA showed significantly (^*∗∗∗*^*p* < 0.001) higher number of neural cells in CA3 region when compared with the same in age matched normal control and saline control group of rats. Additionally, significantly higher number [^a, b^*p* < 0.05] of hippocampal neural cells were observed in CA3 subregion when compared with the same in age matched rat groups supplemented either choline or DHA alone [Figures 3 and 4].

**Figure 5:**
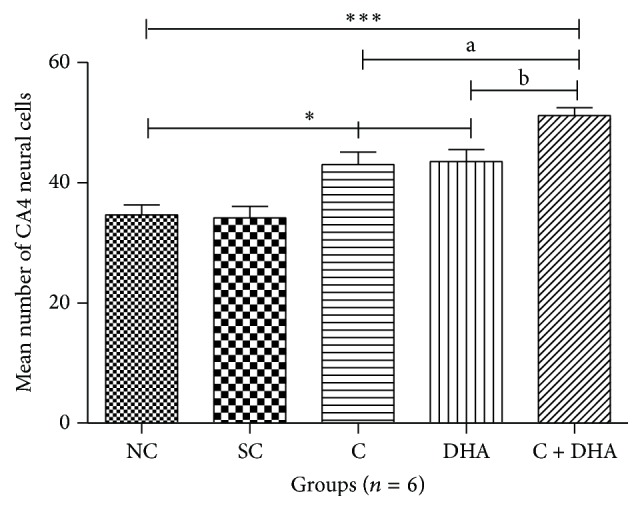
Comparison of neural cells in CA4 region of 5 *μ* thick hippocampal sections. Mean number of neural cells ± Standard Error from 250 *μ*m area of CA4 region of hippocampus from either side in normal control (NC), saline control (SC), choline (C), docosahexaenoic acid (DHA), and choline + docosahexaenoic acid (C + DHA) age matched group of rats. One-way ANOVA followed by Tukey's post hoc test. “*∗*”, NC versus supplemented groups; “a”, C supplemented group versus C + DHA group; and “b”, DHA supplemented group versus C + DHA group. ^*∗*/a/b^*p* < 0.05  &  ^*∗∗∗*^*p* < 0.001.

**Figure 6:**
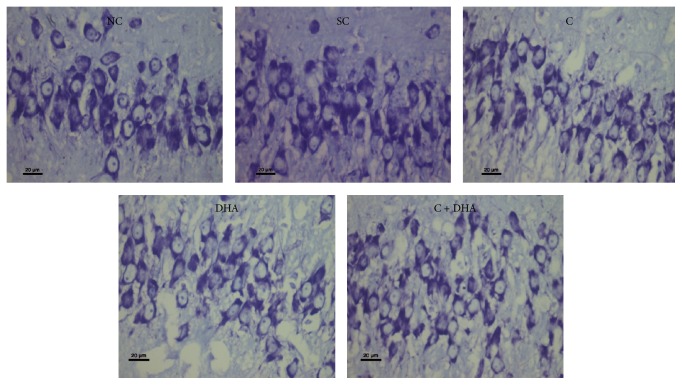
CA4 region: representative photomicrographs of CA4 region from 5 *μ* thick hippocampal sections viewed by 40x magnification, showing Cresyl Violet stained neurons from all experimental groups of PND 40 rats. Note: rats supplemented with combined choline and DHA show significant increase in the number of neural cells in CA4 region.

PND 40 rats supplemented prenatally with either choline or DHA alone shows significant (^*∗*^*p* < 0.05) increase number of neural cells in the CA4 region of hippocampus when compared with the same in age matched normal control and saline control group of rats. However, PND 40 rats supplemented prenatally with combined choline + DHA showed significantly (^*∗∗∗*^*p* < 0.001) higher number of neural cells in the CA4 region of hippocampus when compared with the same in age matched normal control and saline control group of rats. Additionally, significantly higher number [^a, b^*p* < 0.05] of hippocampal neural cells were observed in CA4 subregion when compared with the same in age matched rat groups supplemented with either choline or DHA alone [Figures 5 and 6].

**Figure 7:**
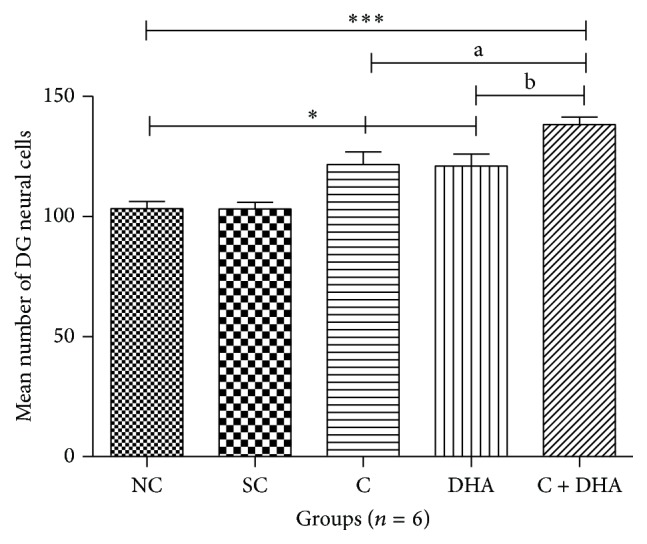
Comparison of number of neural cells in DG region of 5 *μ* thick hippocampal sections. Mean number of neural cells ± Standard Error from 250 *μ*m area of DG region of hippocampus from either side in normal control (NC), saline control (SC), choline (C), docosahexaenoic acid (DHA), and choline + docosahexaenoic acid (C + DHA) age matched group of rats. One-way ANOVA followed by Tukey's post hoc test. “*∗*”, NC versus supplemented groups; “a”, C supplemented group versus C + DHA group; and “b”, DHA supplemented group versus C + DHA group. ^*∗*/a/b^*p* < 0.05  &  ^*∗∗∗*^*p* < 0.001.

**Figure 8:**
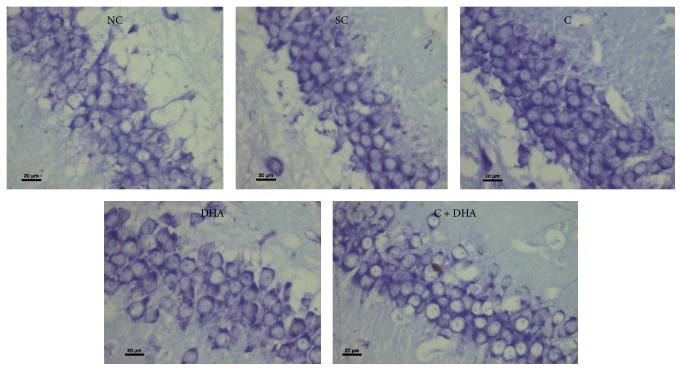
DG region: representative photomicrographs of DG region from 5 *μ* thick hippocampal sections viewed by 40x magnification, showing Cresyl Violet stained neurons from all experimental groups of PND 40 rats. Note: rats supplemented with combined choline and DHA show significant increase in the number of neural cells in DG region.

PND 40 rats supplemented prenatally with either choline or DHA alone show significant (^*∗*^*p* < 0.05) increase in the number of DG neural cells when compared to the same in age matched normal control and saline control group of rats. However, PND 40 rats supplemented prenatally with combined choline + DHA showed significantly (^*∗∗∗*^*p* < 0.001) higher number of DG neural cells when compared with the same in age matched normal control and saline control group of rats. Additionally, significantly higher number [^a, b^*p* < 0.05] of DG neural cells were observed when compared to the same in age matched rat groups supplemented with either choline or DHA alone [Figures 7 and 8].

## 5. Discussion

The results of the present study showed that PND 40 rats supplemented prenatally with choline alone showed significant increase in the number of hippocampal neural cells in the following subregions: CA1, CA3, CA4, and DG when compared with the same in age matched normal control and saline control group of rats. This result is consistent with previous studies that show rodents receiving choline supplements resulting in alterations in hippocampus [20, 22]. Dietary choline content during brain development influences the neurodevelopment of hippocampus and, thus, choline supplementation during pregnancy elicits a major improvement in memory performance of the offspring [30]. In the present study, choline was supplemented during the entire gestational period. A study conducted by Craciunescu et al. in 2003 shows that supplementing choline during embryonic days 12–17 enhances mitosis of the progenitor cell in developing hippocampus [22]. Choline is an essential nutrient and is a precursor for many important compounds, such as acetylcholine, phospholipids, and the methyl donor betaine. Perinatal supplementation of choline in rodents enhances spatial and temporal cognition, which continues across the lifespan [31]. Thus, there may be high demand for choline during prenatal and neonatal periods which stimulates rapid neurogenesis and synaptogenesis [32, 33]. Various studies indicate that choline deficiency during pregnancy affects cognitive function in offspring as prenatal choline availability enhances the attention and spatial memory of the offspring [34]. The need for choline is increased during lactation as it is found that human milk has 1.5 to 2 mM choline moiety per liter [35]. Plasma choline concentrations in humans and other mammals are much higher at birth than those in adults [36].

Additionally in the present study, PND 40 group of rats prenatally supplemented with DHA also shows a significant increase in the number of hippocampal neural cells in the following subregions: CA1, CA3, CA4, and DG, when compared to the same in age matched normal control and saline control group of rats. Studies show that DHA availability significantly alters neurodevelopment in hippocampus and synaptic function. Furthermore, DHA deficiency during development also resulted in marked decreases of synapsins and NMDA receptor subunit NR2A, particularly in CA3 region of hippocampus with concomitant impairment of long-term potentiation [37]. Reduced DHA is interconnected with impairments in behavioral and cognitive function [38].

DHA uptake by the brain depends upon serum levels of DHA and its precursors, which depend upon both dietary intake and liver biosynthesis. Dietary content of DHA markedly influences brain DHA content [39]. DHA deficiencies during gestation alter the neurodevelopment and can lead to later disease and brain malfunction [40]. DHA deficiency during fetal development leads to decreased DHA levels in neural tissue and is associated with deficits in psychomotor development [41], reading skills [42], visual acuity [43], problem solving [44], and attention [45].

Importantly, results of the present study also show that PND 40 rats supplemented prenatally with both choline + DHA showed significantly higher number of hippocampal neural cells in all subregions that were assessed and analyzed [CA1, CA3, CA4, and DG] when compared to the same in age matched normal control and saline control group of rats. Moreover, importantly, PND 40 rats supplemented prenatally with both choline + DHA persistently showed significantly higher number of hippocampal neural cells in all subregions [CA1, CA3, CA4, and DG] when compared to the same in age matched rat groups given either choline or DHA alone. Thus, it is evident that combined supplementation of both choline and DHA is more efficacious in enhancing hippocampal neurodevelopment than supplementing these nutrients separately.

DHA metabolism and choline metabolism are linked by the enzyme phosphatidylethanolamine-N-methyltransferase (PEMT) [12], which catalyzes de novo biosynthesis of phosphatidylcholine (PtdCho) by methylation of PtdEtn enriched with DHA [46]. This enzyme prefers species of PtdEtn that contain long-chain polyunsaturated fatty acids such as DHA [47], thereby forming DHA-enriched species of PtdCho in membranes especially in the brain. Available PtdCho and PtdEtn are also used to form phosphatidylserine (PtdSer) by serine base exchange enzymes [48]. Thus, availability of both choline and DHA during neurodevelopment allows for increased laying down of membranes, a prerequisite for increased neural cell formation in all subregions of hippocampus and dentate gyrus.

## 6. Conclusion

The present study demonstrates the combined efficiency of supplementation with choline and DHA in diet on hippocampal neurodevelopment. The study also emphasizes the requirements of both choline and DHA for better enhancement of fetal brain developmental rather than individual supplementation of any one of the above stated dietary nutrients.

## Figures and Tables

**Table 1:** Rat pellet feed contents obtained from VRK Nutritional Solutions, Pune, having the following ingredients.

Number	Contents	% values
1	Crude protein	21.85%
2	Crude fat	4.85%
3	Crude fibre	3.15%
4	Calcium	1.10%
5	Phosphorus	0.51%
6	Total ash	5.80%
7	Carbohydrates	65.00%

**Table 2:** 

Number	Fatty acids	Diet (g/100 g)
1	C 16:1	0.81
2	C 18:0	0.17
3	C 18:1	0.67
4	C 18:2	2.03
5	C 18:3	0.16
6	C 20:1	0.12

7	Total	3.96
